# *In silico* identification and analysis of paralogs encoding enzymes of carbohydrate metabolism in *Drosophila melanogaster*

**DOI:** 10.17912/micropub.biology.001425

**Published:** 2025-02-05

**Authors:** Rossana Zaru, Steven J Marygold

**Affiliations:** 1 FlyBase, Department of Physiology, Development and Neuroscience, University of Cambridge, Cambridge, U.K.

## Abstract

The identification and characterization of gene paralogs is crucial to understand the functional contribution of individual genes/proteins to biological pathways. Here, we have identified 51 genes belonging to fifteen paralogous groups encoding enzymes involved in carbohydrate metabolism in *Drosophila melanogaster* . Strikingly, most paralogous groups comprise a single ‘canonical' enzyme that is expressed ubiquitously and one or more variants expressed predominantly in the testis. Most of these testis-specific forms are predicted to be catalytically inactive, suggesting they may have adopted regulatory roles. This work will aid the planning and interpretation of experimental studies of several Drosophila metabolic pathways, including glycolysis, gluconeogenesis and the pentose phosphate pathway.

**Figure 1. Paralogs encoding enzymes of simple carbohydrate metabolism in Drosophila .**
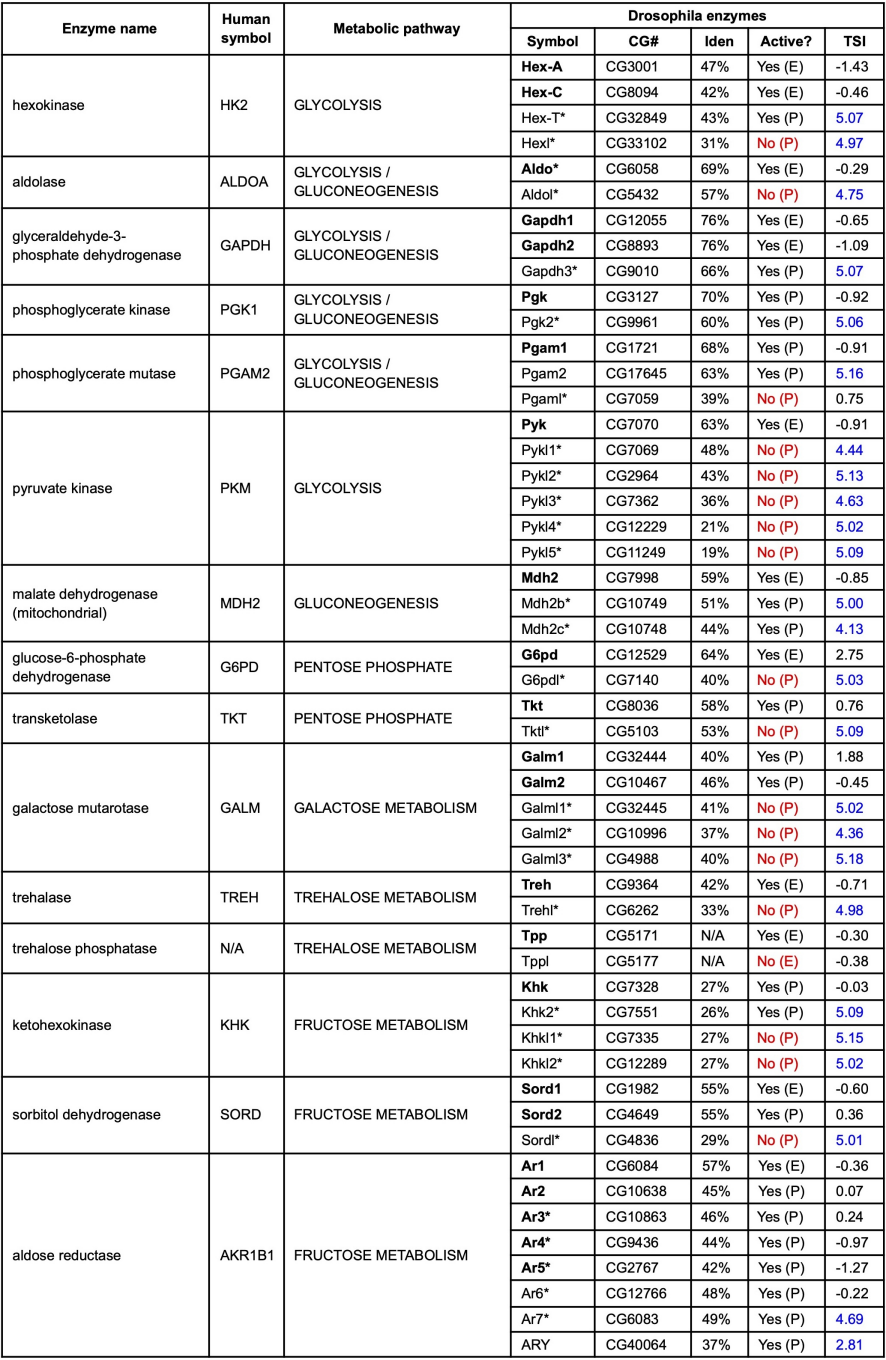
Human symbol: HGNC symbol for the human gene encoding the enzyme most similar to the Drosophila gene(s); Metabolic pathway: major pathway(s) in which the enzyme acts; Symbol: current or new (*) symbol for the Drosophila gene in FlyBase - canonical genes are in bold type; CG#: gene model annotation number in FlyBase; Iden: % amino acid identity between the Drosophila protein and the given human enzyme; Active?: an assessment of whether the enzyme is catalytically active or not, based either on experimental data (E) or predicted from sequence analyses (P) - catalytically inactive pseudoenzymes are indicated in red; TSI: testis-specificity index, which ranges from -2.52 (underrepresented in testis) to 5.2 (very high testis bias) - testis-enriched genes are indicated in blue.

## Description

The biochemical pathways governing the metabolism of simple carbohydrates are highly conserved and critical to life (reviewed by Chandel 2021). For example, glycolysis is a series of enzymatic reactions that catabolize glucose to generate ATP, NADH and pyruvate, the latter of which can be used to fuel energy production or anabolic pathways. Gluconeogenesis is the process of regenerating glucose molecules from non-carbohydrate precursors. This pathway uses many of the same enzymes as glycolysis together with additional enzymes that bypass irreversible glycolytic reactions. The pentose phosphate pathway is an alternative way to oxidize glucose that generates NADPH and ribose-5-phosphate, which are used in many important biosynthetic reactions. Additional pathways metabolize other simple sugars, such as fructose, galactose and trehalose.

We are currently reviewing and annotating the carbohydrate metabolic pathways of *Drosophila melanogaster* (hereafter, Drosophila). In so doing, we have identified fifteen instances of duplicate genes (paralogs) encoding a total of 51 enzymes predicted to metabolize simple sugars (Table 1). Most paralogous groups comprise two or three members, though there are five galactose mutarotase genes, six pyruvate kinase genes (Heidarian et al. 2023) and eight paralogs encoding aldose reductase.

Each group has at least one ‘canonical' gene, defined as a paralog with widespread expression in somatic tissues and whose encoded protein is known/predicted to be catalytically active (shown in bold text in Table 1; also see Extended Data Files). Most of these 23 genes have been identified previously and are named using standard enzyme nomenclature, including a distinguishing alpha-numerical suffix where necessary. Five groups contain multiple canonical genes. In four of these cases, the paralogs exhibit some qualitative and/or quantitative differences in their expression: *Hex-A* is expressed in most tissues throughout development whereas *Hex-C* has peak expression in the fat body and digestive system of adults (Duvernell and Eanes 2000) ; *Galm1* expression is high in most adult tissues, whereas *Galm2* is generally expressed at lower levels and mainly in the digestive system of adults (Öztürk-Çolak et al. 2024); *Sord1* expression is higher than *Sord2* across all tissues (Luque et al. 1998) ; and *Ar2* is expressed at distinctly lower levels compared to *Ar1, Ar3* , *Ar4* and *Ar5* (Öztürk-Çolak et al. 2024). In contrast, the two *Gapdh* paralogs show near identical expression profiles across different tissues and developmental stages (Öztürk-Çolak et al. 2024).

The 28 ‘non-canonical' paralogs are characterized by their sequence divergence from the canonical gene(s), predicted lack of catalytic activity in their encoded protein, and/or tissue-restricted expression pattern (Table 1; also see Extended Data Files). Most are identified, analyzed and named here for the first time. 18 of these genes are known or predicted to encode catalytically inactive proteins owing to loss of critical residues in active sites and/or substrate-binding sites (Yoshida et al. 2016; Duvernell et al. 2000; see Extended Data File 1). Such genes are therefore considered to encode pseudoenzymes and are referred to using the standard enzyme nomenclature with a ‘like' suffix, adding a distinguishing numerical suffix where necessary. For example, the *Transketolase* ( *Tkt* ) gene encodes the canonical enzyme, whereas *Transketolase like* ( *Tktl* ) is a divergent paralog predicted to encode a pseudoenzyme. Strikingly, the expression of almost all these non-canonical paralogs is restricted to the testis. This is evident by inspecting their ‘testis specificity index', where higher scores indicate testis-restricted expression (Vedelek et al. 2018; Table 1). It is possible that these duplicates have diverged to meet distinct metabolic demands of the testis environment, functioning either as tissue-specific enzymes or as non-catalytic modulators of canonical enzymes. Significantly, several of the Drosophila testis-specific paralogs have human counterparts with testis-enriched expression, including *HK1* , *GAPDHS* , *PGK2* , *TKTL1* , *TKTL2* and AKR1E2 (UniProt Consortium 2023) .

The paralogs mentioned herein arose in the Drosophila genome through different evolutionary means (see Extended Data File 1). Several paralogs have been classed as ‘retrogenes', meaning that they originated via retrotransposition from a parental gene copy, namely: *Gapdh1* , *Pgam2* , *Mdh2b* or *Mdh2c* , *G6pdl* , *Galm2* , *Galml2* and *Ar5* (Currie and Sullivan 1994; Dai et al. 2006; Bai et al. 2007; Langille and Clark 2007; Pan and Zhang 2009). Other gene pairs are directly adjacent in the genome and therefore probably arose by a tandem duplication event. These are: *Hex-T* / *Hexl* ; *Pgk* / *Pgk2* ; *Pyk* / *Pykl1* ; *Mdh2b* / *Mdh2c* ; *Galm1* / *Galml1* ; *Tpp* / *Tppl* ; *Khk* / *Khkl1* ; *Khk2* / *Khkl2* ; *Ar1* / *Ar7* and *Ar3* / *Ar6* (Duvernell and Eanes 2000; Heidarian et al. 2023; Öztürk-Çolak et al. 2024). The remaining paralogs likely derived from other types of gene duplication event (Reams and Roth 2015) .

In summary, we find that many genes encoding enzymes governing carbohydrate metabolism are duplicated in Drosophila. Several of these paralogs display tissue-specific, notably testis-specific, expression, and a third are predicted to encode pseudoenzymes. This information is critical to experimental studies of these genes and, as such, will be integrated into FlyBase and allied databases in the form of updated gene nomenclature and Gene Ontology annotations.

## Methods

Drosophila genes encoding enzymes involved in carbohydrate metabolic pathways were identified using an integrative approach combining Gene Ontology (GO) annotation data at FlyBase (Öztürk-Çolak et al. 2024) with computed pathway resources at FlyCyc (Caspi et al. 2020) , Reactome (Milacic et al. 2024) and KEGG (Kanehisa et al. 2024) . Fly gene and protein data were obtained from FlyBase (http://flybase.org) version FB2024_06. Paralogous genes, human-Drosophila orthologs and amino acid identities were determined using the implementation of DIOPT (Hu et al. 2011) within FlyBase. The testis-specificity index (TSI) for each gene was originally computed from modENCODE RNAseq data by Vedelek et al. (2018) and was retrieved from the TSI implementation in FlyBase. High-throughput expression data shown in Extended Data File 2 were derived from the implementation of FlyAtlas 2 (Leader et al. 2018) within FlyBase. The potential catalytic activity of uncharacterized Drosophila enzymes was assessed by using the UniProt ‘Align' tool to align their protein sequences with those of characterized (usually human) orthologs and manually inspecting conservation of critical residues at the annotated active site and substrate/cofactor binding sites (Zaru et al. 2023) .
